# Comparison of clinical and pathological features between early-stage gastric-type and intestinal-type differentiated adenocarcinoma: a retrospective study

**DOI:** 10.1186/s12876-023-02733-3

**Published:** 2023-03-28

**Authors:** Borui Li, Tingting Chen, Dingbao Liang, Yin Zhang, Xiwei Ding, Ying Lv

**Affiliations:** 1 Department of Gastroenterology, Nanjing Drum Tower Hospital, The Affiliated Hospital of Nanjing University Medical School, Nanjing, China; 2 Department of Pathology, The Affiliated Drum Tower Hospital of Nanjing University Medical School, Nanjing, China; 3 Department of Gastroenterology, Navy Anqing Hospital, Anqing, China; 4 Department of Gastroenterology, Nanjing Drum Tower Hospital, Clinical College of Nanjing Medical University, Nanjing, China

**Keywords:** Gastric-type differentiated adenocarcinoma, Intestinal-type differentiated adenocarcinoma, Mucin phenotype

## Abstract

**Background:**

The clinicopathological features and endoscopic characteristics under magnifying endoscopy with narrow band imaging (ME-NBI) between early-stage gastric-type differentiated adenocarcinoma (GDA) and intestinal-type differentiated adenocarcinoma (IDA) remain controversial.

**Methods:**

Early gastric adenocarcinomas that underwent endoscopic submucosal dissection (ESD) in Nanjing Drum Tower Hospital between August 2017 and August 2021 were included in the present study. GDA cases and IDA cases were selected based on morphology and immunohistochemistry staining of CD10, MUC2, MUC5AC, and MUC6. Clinicopathological data and endoscopic findings in ME-NBI were compared between GDAs and IDAs.

**Results:**

The mucin phenotypes of 657 gastric cancers were gastric (*n* = 307), intestinal (*n* = 109), mixed (*n* = 181) and unclassified (*n* = 60). No significant difference was observed in terms of gender, age, tumor size, gross type, tumor location, background mucosa, lymphatic invasion, and vascular invasion between patients with GDA and IDA. GDA cases were associated with deeper invasion than IDA cases (*p* = 0.007). In ME-NBI, GDAs were more likely to exhibit an intralobular loop patten, whereas IDAs were more likely to exhibit a fine network pattern. In addition, the proportion of none-curative resection in GDAs was significantly higher than that in IDAs (*p* = 0.007).

**Conclusion:**

The mucin phenotype of differentiated early gastric adenocarcinoma has clinical significance. GDA was associated with less endoscopically resectability than IDA.

## Introduction

Gastric carcinoma (GC), one of the most common malignancies in the world, is known as a kind of heterogeneous disease displaying a variety of phenotypes with different prognostic characteristics. Histologically, gastric cancer has been divided into two major categories, intestinal and diffuse types according to Lauren classification system [1], or differentiated and undifferentiated types according to Nakamura classification system based on glandular structure [2]. According to the latest classification released by Japanese Gastric Cancer Association (JGCA) in 2017, gastric cancer is generally classified into five major types: papillary, tubular, poorly differentiated, mucinous, and signet-ring cell [3]. Among different types, papillary adenocarcinoma (pap), well differentiated tubular adenocarcinoma (tub1), and moderately differentiated tubular adenocarcinoma (tub2) belong to differentiated GC. Poorly differentiated, mucinous, and signet-ring cell belong to undifferentiated GC. The histological type is determined according to its predominant component. Therefore, if the proportion of differentiated components in the lesion is greater than or equal to 50%, it is regarded as differentiated gastric cancer. Otherwise, it is regarded as undifferentiated gastric cancer.

**Fig. 1:**
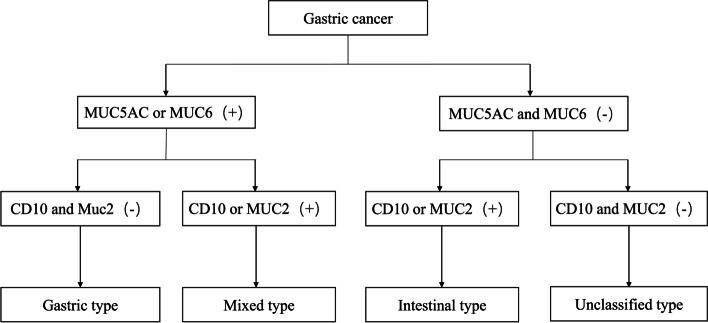
Classification of gastric cancer based on mucous expression

Mucin is a heavily glycosylated glycoprotein produced by cancers [4]. With increasing research on mucin and the progress of immunohistochemical studies, a mucin phenotype subclassification come into sight, independent of histologic type. Differentiated gastric adenocarcinomas can be classified as having either gastric, intestinal, mixed or unclassified mucin phenotype based on the expression of MUC5AC (a marker of foveolar cells), MUC6 (a marker of gastric mucous neck cells and pyloric glands), MUC2 (a marker of intestinal goblet cells), and CD10 (a marker of the brush border of the small intestinal absorptive cells) (Fig. 1).

Accumulating evidence has indicated that mucin phenotype correlated with the development pattern and malignant degree of gastric adenocarcinoma. Several studies indicated that the gastric phenotype had a higher potential for lymph node metastasis, deeper invasion depth, and correlated with a worse prognosis than the intestinal type [5–7]. On the contrary, Kim et al. indicated that the gastric phenotype was linked to a better prognosis [8]. Most of the above studies investigated advanced GCs and did not focus on early GCs. Whether early gastric-type differentiated adenocarcinoma (GDA) have more malignant potential than early intestinal-type differentiated adenocarcinoma (IDA) remains controversial due to insufficient data.

Magnifying endoscopy with narrow band imaging (ME-NBI) is an effective method for the detailed visualization of microsurface and microvascular structures within the superficial layer of the gastric mucosa. Recent studies showed that the ME-NBI is able to predict histological characteristics of early gastric cancers [9–13]. Two studies have also reported that mucin phenotype was significantly correlated with ME-NBI results in early gastric cancers. However, the results in these studies were not consistent. In addition, the sample size of these studies was small.

It is important to characterize the clinicopathological and endoscopic differences between early GDAs and IDAs in order to clarify the clinical significance of mucin phenotype. In this study, we investigated the clinicopathological and endoscopic features of early GDAs in comparison with early IDAs. We also compared the endoscopic curability between GDAs and IDAs based on the Japanese gastric cancer treatment guideline. To the best of our knowledge, the present study is the largest study, conducted to date, evaluating the significance of mucin phenotype of early differentiated gastric adenocarcinoma [5, 8, 14–16].

## Methods

### Patients

Over the period from August 2017 to August 2021, a total of 715 patients with early gastric cancer were treated with endoscopic submucosal dissection (ESD) at Nanjing Drum Tower Hospital affiliated to Nanjing University Medical School. All the patients had absolute indications for ESD and were examined with ME-NBI before resection. Histologic results were classified according to the JGCA. In this study, patients with differentiated gastric type adenocarcinoma and intestinal type adenocarcinoma were retrospectively included. The exclusion criteria was undifferentiated adenocarcinoma, adenocarcinoma with combined mucin phenotype, and adenocarcinoma with unclassified mucin phenotype. Gastric adenocarcinomas of fundic gland type were also excluded because they were unique subtypes of GDA with good prognosis [17].

This study was approved by the institutional review board of the Ethics Committee of Nanjing Drum Tower Hospital affiliated to Nanjing University Medical School (IRB no.2022–717-01) and was performed according to the Declaration of Helsinki. The informed consent was waived by the Ethics Committee of Nanjing Drum Tower Hospital due to the retrospective design of the study.

### Immunohistochemistry and classification of mucin phenotype

All the resected specimens were fixed with 10% buffered formalin and embedded in paraffin. The 4-µm-thick consecutive sections were used for histologic examination by hematoxylin and eosin staining and immunohistochemistry. D2–40 staining (Dako,1:200) was used in all cases to assess lymphatic invasion and CD-31 staining (Zsbio,1:200) to assess vascular invasion. Immunohistochemical staining was performed according to the standard methods using antibodies against MUC5AC (MRQ-19, 1:200, Zsbio), MUC2 (Ccp58, 1:200, Zsbio), MUC6 (MRQ-20, 1:200, Zsbio), and CD10 (56C6, 1:200, Zsbio). Immunostaining was regarded positive for the relevant marker if more than 10% of the cancer cells was distinct staining as previously reported [18–23]. The gastric type GC exhibited positive staining for at least one of the MUC5AC and MUC6, while negative for CD10 and MUC2. The intestinal type GC showed positive staining for at least one of the CD10 and MUC2, while negative for MUC5AC and MUC6. Mixed-type tumors were defined as tumors having immunostaining consistent with the gastric phenotype (positive cells for MUC5AC and/or MUC6) as well as the intestinal phenotype (positive cells for CD10 and/or MUC2). Finally, tumors without immunostaining for either gastric or intestinal markers were defined as unclassified phenotype. Pathological factors such as tumor size, depth of invasion, location, lymphatic or vascular invasion, histologic type were reviewed according to the JCGA [3].

### ME-NBI

The instruments used in our study were an electronic endoscopic system (EVIS LUCERA Spectrum; Olympus, Tokyo, Japan) and a high resolution magnifying video endoscope (GIF-H260Z and GIF-H290Z, Olympus, Tokyo, Japan). A soft hood was mounted at the tip of the endoscope to obtain a clear view for ME-NBI. Microvessel patterns were classified into intralobular loop pattern (ILL), fine network patten (FNP), and corkscrew patten (CSP) according to previous studies [9, 12, 13]. If more than one pattern were observed in the lesion, the lesion was classified based on the predominant vascular pattern.

### Endoscopic curability

In the latest Japanese ESD Guidelines for early gastric cancer, endoscopic curability of early GC was divided into three grades: eCuraA, eCuraB and eCuraC. Grade C was subdivided into C1 and C2 [24, 25]. The eCuraA classification must meet all the following conditions: en bloc resection, tumor confined to the mucosa, no lymphovascular invasion, negative results for horizontal margin and vertical margin. Moreover, when no ulcerative area spotted in the tumor, histologically differentiated dominant tumor of any size and histologically undifferentiated dominant tumor that is less than or equal to 2 cm are classified as eCuraA. However, the endoscopic curability will be classified as eCuraC-2 if the size of undifferentiated type-dominant lesion is more than 2 cm. Provided that the ulcerative area was spotted in the tumor, but the size of histologically differentiated dominant tumor is less than or equal to 3 cm, the endoscopic curability is also classified as eCuraA. The eCuraB classification must meet all the following conditions: en bloc resection, histologically of differentiated type-dominant, tumor invasion is within 0.5 mm of the muscularis mucosae, tumor size ≤ 3 cm, no lymphovascular infiltration, negative results for horizontal margin and vertical margin. However, if the submucosal lesion contains undifferentiated components, the endoscopic curability will be classified as eCuraC-2. When the tumor is histologically differentiated type-dominant, either not completely resected or the horizontal margin being positive, the endoscopic curability will be classified as eCuraC-1, even if other criteria for eCuraA or eCuraB is fulfilled. When the resection does not meet the conditions of eCuraA, eCuraB and eCuraC-1, all other resections will be classified as eCuraC-2. eCuraC corresponds to the concept of non-curative resection which usually need additional treatment. In current study, we compared the degree of curability between GDAs and IDAs using eCura grades based on ESD pathology result.

### Statistical analysis

The chi-square test or Fisher’s exact test were used to compare proportions between groups. Student’s t test or the Mann–Whitney U test was used to compare groups in terms of age and tumor size. *P* value < 0.05 was considered significant.

## Results

### Study sample

**Fig. 2:**
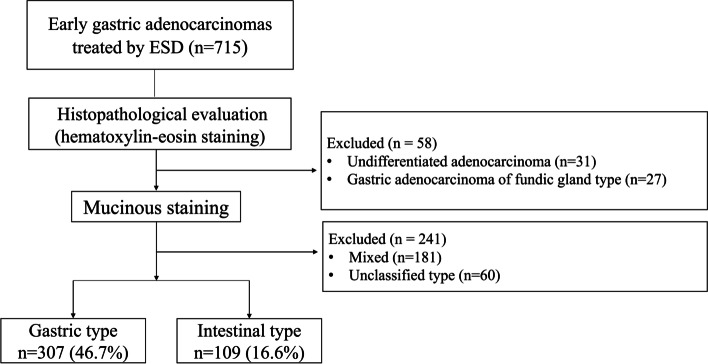
Flowchart illustrating the inclusion and exclusion of lesions in the study

The flowchart of the current study is shown in Fig. 2. Of all the 715 lesions, 31 undifferentiated adenocarcinomas and 27 gastric adenocarcinomas of fundic gland type were excluded according to the study design. The expression of MUC5AC, MUC6, MUC2, and CD10 was shown in 346 (52.7%), 425 (64.7%), 149 (22.7%), and 176 (26.8%) of the 657 early differentiated gastric cancer lesions, respectively. Based on the expression of these markers, 307 (46.7%) were classified as gastric phenotype tumors, 109 (16.6%) as intestinal phenotype tumors, 181 (27.5%) as mixed phenotype tumors, and 60 (9.1%) as unclassified tumors. Undifferentiated components were observed in 33 (10.7%) lesions among 307 gastric phenotype tumors, while observed in 6 (5.5%) lesions among 109 intestinal phenotype tumors. The difference was not statistically significant (*p* = 0.107).

### Clinicopathologic characteristics

In this study, 307 GDAs and 109 IDAs were included for further comparison of clinicopathological features. As shown in Table 1, no significant difference was seen in terms of gender, age, tumor size, gross type, tumor location, lymphatic invasion and vascular invasion between patients with GDA and IDA. In comparison with tub1, tub2 and pap were more malignant differentiated adenocarcinomas. Although the proportion of tub2 and pap were higher in GDA group than that in IDA group, the difference was not statistically significant (23.8% versus 13.7%, *p* = 0.088). Among the 307 cases of GDA, intramucosal lesions, SM1 lesions and SM2 lesions were observed in 236 (76.9%), 40 (13%), and 31 (10.1%) cases, respectively. Among the 109 cases of IDA, intramucosal lesions, SM1 lesions and SM2 lesions were observed in 99 (90.8%), six (5.5%), and four (3.7%) cases, respectively. Chi-squared test showed GDA cases were associated with deeper invasion than IDA cases (*p* = 0.007). In peritumoral region, the rate of atrophic gastritis and intestinal metaplasia were similar in both groups (*p* = 0.478 and *p* = 0.447).

**Table 1:** Comparison of the clinicopathological characteristics between gastric-type differentiated adenocarcinoma and intestinal-type differentiated adenocarcinoma

	GDA (*n* = 307)	IDA (*n* = 109)	*p* value
Patient characteristics			
Age (years), mean ± SD	65.0 ± 9.4	65.3 ± 8.5	0.898
Gender (male/female)	233/74	80/29	0.603
Pathological diagnosis			
Tumor size(mm), mean ± SD	19.9 ± 13.3	18.2 ± 10.8	0.557
Histology, n (%)			0.088
Tub1	234 (76.2)	94 (86.2)	
Tub2	67 (21.8)	14 (12.8)	
Pap	6 (2.0)	1 (0.9)	
Depth of invasion, n (%)			0.007
M	236 (76.9)	99 (90.8)	
SM1	40 (13.0)	6 (5.5)	
SM2	31 (10.1)	4 (3.7)	
Lymphatic invasion, n (%)			1
Positive	4 (1.3)	1 (0.9)	
Negative	303 (98.7)	108 (99.1)	
Vascular invasion, n (%)			1
Positive	1 (0.3)	1 (0.9)	
Negative	306 (99.7)	108 (99.1)	
Incisal margin			1
Positive	3 (1.0)	1 (0.9)	
Negative	304 (99.0)	108 (99.1)	
Basal margin			0.930
Positive	8 (2.6)	4 (3.7)	
Negative	299 (97.4)	105 (96.3)	
Ulcer			0.798
Positive	15 (4.9)	2 (1.8)	
Negative	292 (95.1)	107 (98.2)	
Macroscopic type			0.124
Elevated	104 (33.9)	46 (42.2)	
Flat	77 (25.1)	30 (27.5)	
Depressed	126 (41.0)	33 (30.3)	
Macroscopic findings, n (%)			
Location			0.278
Upper third	104 (33.9)	34 (31.2)	
Middle third	47 (15.3)	24 (22.0)	
Lower third	156 (50.8)	51 (46.8)	
Background mucosa			0.478
Atrophic	257 (83.7)	88 (80.7)	
Non-atrophic	50 (16.3)	21 (19.3)	
Background mucosa			0.447
Intestinal metaplasia	255 (83.1)	87 (79.8)	
Non-intestinal metaplasia	52 (16.9)	22 (20.2)	

### Microvessel patterns

**Fig. 3:**
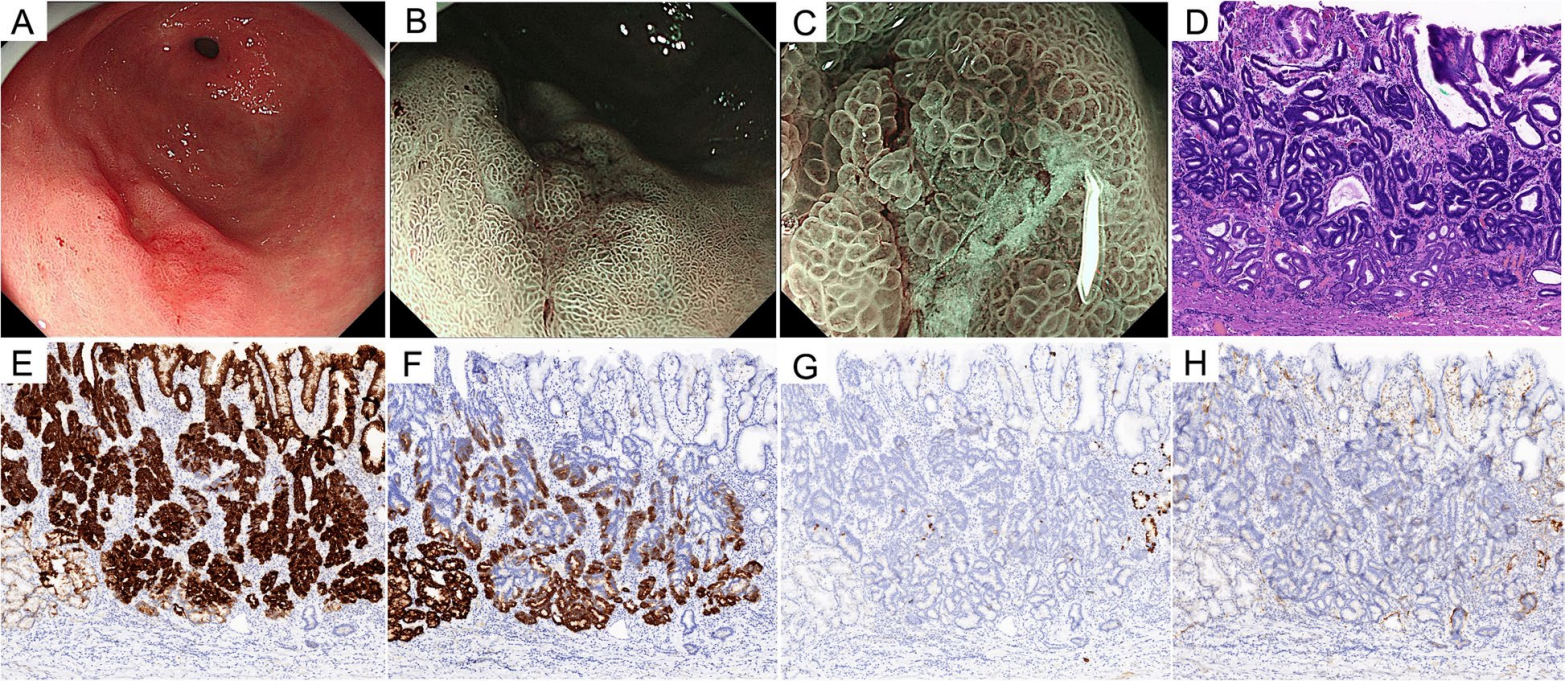
A case of gastric-type differentiated cancer. **A** A depressed lesion is observed in the lower third of body. **B** Magnifying endoscopy with narrow band imaging indicates a tubular microsurface pattern. **C** Magnifying endoscopy with narrow band imaging indicates an intralobular loop microvascular pattern. **D** Histologic examination shows a tubular 1 (well-differentiated) adenocarcinoma in the lamina propria (H&E stain, × 100). The cancer cells are strongly positive for MUC5AC (**E**) and MUC6 (**F**), negative for MUC2 (**G**) and CD10 (**H**)

**Fig. 4:**
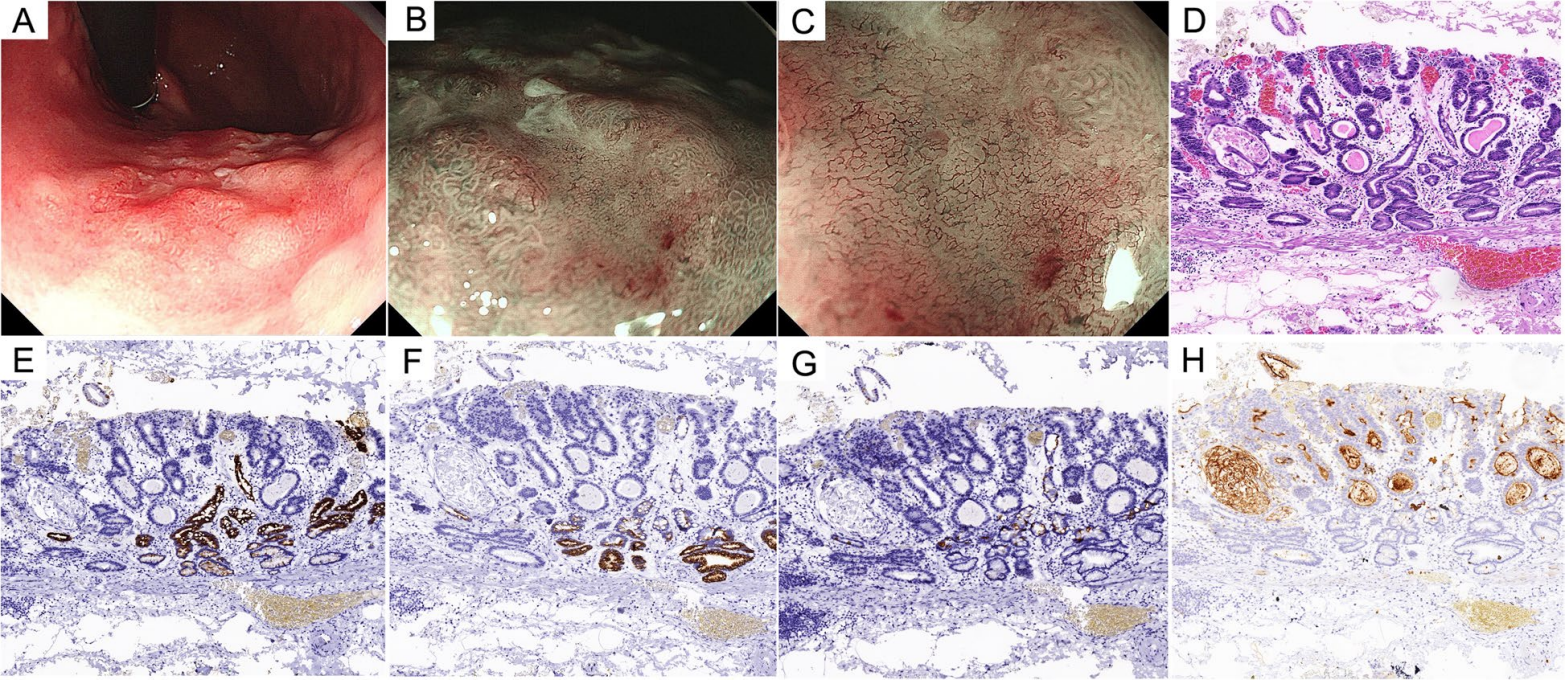
A case of intestinal-type differentiated cancer. **A** A flat lesion is observed in the middle third of body. **B** Magnifying endoscopy with narrow band imaging indicates a absent microsurface pattern. **C** Magnifying endoscopy with narrow band imaging indicates a fine network microvascular pattern. **D** Histologic examination shows a tubular 2 (moderately differentiated) adenocarcinoma in the muscularis mucosa (H&E stain, × 100). The cancer cells are strongly positive for CD10 (**E**), negative for MUC5AC (**F**), MUC6 (**G**) and MUC2 (**H**)

The characteristics of the microvascular patterns in GDA and IDA cases are summarized in Table 2. Among GDAs, ILL was seen in 239 lesions (78%), FNP in 64 lesions (21%), and CSP in 4 lesions (1%). Among IDAs, ILL was observed in 51 lesions (47%), FNP in 56 lesions (51%), and CSP in 2 lesions (2%). The main microvessel patterns were ILL in GDAs and FNP in IDAs (*p* < 0.001). Therefore, GDAs were more likely to exhibit an ILL microvessel patten, whereas IDAs were more likely to exhibit a FNP microvessel pattern. Representative pictures of a GDA and IDA were shown in Figs. 3 and 4.

**Table 2:** Comparison of the microvascular patterns between gastric-type differentiated adenocarcinoma and intestinal-type differentiated adenocarcinoma

	GDA (*n* = 307)	IDA (*n* = 109)	*p* value
Microvascular pattern			< 0.001
Intralobular Loop	239 (77.9)	51 (46.8)	
Fine network	64 (20.8)	56 (51.4)	
Corkscrew	4 (1.3)	2 (1.8)

### Evaluation of endoscopic curability and follow-up

Comparisons of the endoscopic curability between GDA and IDA are shown in Table 3. In line with the Japanese gastric cancer guideline, 221 (71.9%) GDA cases were classified as eCura-A, 22 (7.2%) were classified as eCura-B, seven (2.3%) were classified as eCura-C1, and 57 (18.6%) were classified as eCura-C2. Among IDA cases, 96 (88.1%) were classified as eCura-A, three (2.8%) were classified as eCura-B, three (2.8%) were classified as eCura-C1, and 7 (6.4%) were classified as eCura-C2. Therefore, 64 (20.8%) GDA cases were regarded as non-curative resection, while only 10 (9.2%) IDA cases were categorized as non-curative resection. There were significant differences in terms of the curative criteria classification based on the eCura System (*p* = 0.002) and in terms of the proportion of non-curative resection between the two groups (*p* = 0.007). In summary, GDA was associated with lower endoscopic curability than IDA. For non-curative cases, 25 GDA cases and one IDA case underwent additional surgery. Among the 25 GDA cases who underwent additional surgery, one case had residual tumor, and two cases had lymph node metastasis. No residual tumor or lymph node metastasis was found in the one IDA case who received additional surgery. All patients were closely followed up, one (0.33%) GDA patient died of none-cancer specific causes and two (0.65%) GDA patients recurred. All IDA patients survived and one (0.9%) IDA patient recurred. The findings of analysis indicate no significant difference of prognosis between the GDA and the IDA.

**Table 3:** Comparison of the classification of eCura System and evaluation of endoscopic curability between gastric-type differentiated adenocarcinoma and intestinal-type differentiated adenocarcinoma

	GDA (*n* = 307)	IDA (*n* = 109)	*p* value
eCura grade			0.002
eCura A	221 (72.0)	96 (88.1)	
eCura B	23 (7.5)	3 (2.8)	
eCura C1	7 (2.3)	4 (3.7)	
eCura C2	56 (18.2)	6 (5.5)	
Evaluation of endoscopic curability			0.007
Curative resection	244 (79.5)	99 (90.8)	
Non-curative resection	63 (20.5)	10 (9.2)	

## Discussion

In the present study, 657 early differentiated gastric carcinomas were classified as gastric phenotype tumors (307; 46.7%), intestinal phenotype tumors (109; 27.5%), mixed phenotype tumors (181; 27.5%), and unclassified phenotype tumors (60; 9.1%). Previous reports have shown the incidences of gastric, intestinal, mixed, and unclassified phenotype GCs to be 16.3–53.1%, 10.3–35%, 19.4–56% and 0–19.6%, respectively. The percentage of mucin type varied considerably among different studies. One possible reason is that different study uses different definitions of mucin positivity, antibodies, and case groups. We showed that gastric type is the most common mucin phenotype among early differentiated gastric adenocarcinomas [15, 19, 26].

To clarify the significance of the mucin patterns in early differentiated gastric cancer, we analyzed the relation between gastric/intestinal patterns and the clinicopathologic parameters using ESD cases from our hospital. No significant difference was observed in terms of gender, age, tumor size, gross type, tumor location, background mucosa, lymphatic invasion and vascular invasion between patients with GDA and IDA. However, GDA cases were associated with deeper invasion than IDA cases. This may indicate a more biological aggressiveness of GDAs than IDAs.

Differentiated-type-predominant early GC including undifferentiated-type components has been shown to have higher malignancy than pure differentiated-type early GC [27, 28]. Song et al. reported that gastric-type mucin was expressed in mixed-type early GC with a higher frequency, compared with intestinal-type mucin. Gastric-type tumors were associated with a higher proportion of undifferentiated-type tumors [29]. Our study also shows GDA was associated with a higher proportion of undifferentiated components. However, the difference did not reach statistically significance (*p* = 0.107).

Although clinicopathological factors of different mucin phenotype have been reported in many studies, no study has investigated the association between mucin phenotype and endoscopic curability. According to the most recent Japanese gastric cancer ESD guideline, endoscopic curability of early gastric cancer can be divided into eCura A, eCura B, eCura C1, and eCuraC-2 [24]. eCura A and eCuraB have been regarded as curative resection, which are considered as having the same therapeutic effect as surgery. eCura C1 and eCura C2 have been regarded as no-curative resection, needing additional treatment after ESD. In this study, the proportion of no-curative resection in GDAs was significantly higher than that in IDAs, indicating that GDA is less endoscopically resectable than IDA. Valente et al. reported that the depressed type of intramucosal differentiated-type gastric cancer has higher malignant potential in comparison to the elevated type [30]. The GDA showed a higher proportion of depressed lesions and undifferentiated-type components than the IDA, though the difference did not reach statistically significance. The above result may in part illustrate the higher malignant potential of the GDA compared with the IDA. Therefore, endoscopic submucosal dissection for early gastric-type differentiated adenocarcinoma must be carried out with more caution and followed-up more intensively. This is the first study to investigate an association between mucin phenotype and curability of endoscopic resection based on the classification of endoscopic curability in the Japanese gastric cancer guideline.

Previous studies have posited that the GDA contains a higher potential for lymphatic and vascular invasion and lymph node metastasis than the IDA, consequently having worse prognosis [8–10]. In our study, the GDA cases revealed deeper invasion than the IDA cases, but there were little lymphatic and vascular invasion either in the GDA cases or in the IDA cases. Owing to the endoscopic resection, we cannot compare the rate of lymph node metastasis in different groups. Although GDA invaded deeper than IDA, these lesions were still confined to the submucosa, which may explain why the prognosis had no significant difference between gastric type and intestinal type in early gastric cancer.

Since GDA shows more malignant potential, early recognition and diagnosis under endoscopy is quite important. ME-NBI system can predict the histological characteristics of gastric cancer lesions [10]. Yokoyama et al. have shown that ILL and FNP were the main microvessel patterns for most differentiated GCs. This study showed that GDAs mainly displayed an ILL pattern, whereas IDAs demonstrated a higher ratio of FNP pattern. Kobayashi et al. also reported that GC with gastric mucin showed a high ratio of ILL pattern (61.5%) and GC with intestinal mucin showed a high ratio of FNP pattern (66.7%). Our results confirmed these findings, which indicated ILL and FNP were the main microvessel patterns for GDAs and IDAs, respectively. In contrast, OK et al. described that the most common microvessel pattern in gastric type adenocarcinoma was CSP pattern [12]. However, they also included 34.4% undifferentiated adenocarcinomas and 90.9% undifferentiated adenocarcinomas displayed CSP pattern in their study. This may account for the predominance of the CSP pattern in gastric type adenocarcinomas. Study by OK et al. also classified microsurface patterns as tubular/oval, papillary, destructive, and absent, and showed that microsurface patterns did not vary significantly with mucin phenotypes. Our study did not compare the microsurface pattern between different groups due to the following reasons. First, it was subjective to classify the microsurface into destructive and absent pattern. Second, surface glandular structure in some differentiated adenocarcinomas could not be observed clearly without crystal violet or acetic acid staining. In this study, there was a significant difference in the microvascular pattern between GDAs and IDAs. These findings suggest that expression of gastrointestinal mucins may in part help to explain the differences in the vascularization patterns of early differentiated gastric adenocarcinomas. However, it is difficult to use microvascular patterns alone as a meaningful diagnostic tool to predict mucin phenotypes due to the low specificity. Microvessel patterns combined with immunohistochemistry of biopsy specimens prior to ESD may be helpful in differentiating mucin types. Further prospective studies will be needed to verify the clinical significance of microvascular patterns in early differentiated gastric adenocarcinomas.

Our study has some limitations. First, only endoscopically resected early gastric cases were included and early or advanced gastric cancer cases resected by surgery were excluded. The methods of sampling of ESD specimens and surgical specimens may be different and the pathological evaluation of the depth of invasion of surgical specimens is less accurate than that of ESD. Moreover, a great number of surgically resected GDA cases did not undergo preoperative magnifying endoscopy. Second, mixed phenotype and unclassified phenotype were not included because we aimed to compare the clinicopathological features between early gastric-type and intestinal-type differentiated adenocarcinoma. In addition, there is little or no understanding of mixed phenotype and unclassified phenotype, especially in the mechanism of carcinogenesis and development. The underlying processes resulting in these two phenotypes require further research.

## Conclusion

The clinicopathological characteristics of gastric cancer with gastric phenotype differ from those of gastric cancer with intestinal phenotype in terms of invasion depth, microvessel pattens in ME-NBI, and endoscopic resectability.

## Data Availability

The data that support the findings of this study are available from the corresponding author upon reasonable request.

## Abbreviations

ME-NBI: Magnifying endoscopy with narrow band imaging
GDA: Gastric-type differentiated adenocarcinoma
IDA: Intestinal-type differentiated adenocarcinoma
ESD: Endoscopic submucosal dissection
GC: Gastric carcinoma
JGCA: Japanese Gastric Cancer Association
Pap: Papillary adenocarcinoma
Tub1: Well differentiated tubular adenocarcinoma
Tub2: Moderately differentiated tubular adenocarcinoma
ILL: Intralobular loop pattern
FNP: Fine network patten
CSP: Corkscrew patten
